# Corticosteroid co-treatment induces resistance to chemotherapy in surgical resections, xenografts and established cell lines of pancreatic cancer

**DOI:** 10.1186/1471-2407-6-61

**Published:** 2006-03-15

**Authors:** Chengwen Zhang, Armin Kolb, Peter Büchler, Andrew CB Cato, Jürgen Mattern, Werner Rittgen, Lutz Edler, Klaus-Michael Debatin, Markus W Büchler, Helmut Friess, Ingrid Herr

**Affiliations:** 1 Research Group Molecular Urooncology,German Cancer Research Center, Im Neuenheimer Feld 280, 69120 Heidelberg, Germany; 2 Department of General Surgery,University of Heidelberg, Im Neuenheimer Feld 110, 69120 Heidelberg, Germany; 3 Research Center Karlsruhe, Institute of Toxicology and Genetics, H.-v. Helmholtz-Platz 1, 76344 Eggenstein-Leopoldshafen, Germany; 4 Clinical Cooperation Unit Nuclear Medicine,German Cancer Research Center, Im Neuenheimer Feld 280, 69120 Heidelberg, Germany; 5 Department of Biostatistics, German Cancer Research Center, Im Neuenheimer Feld 280, 69120 Heidelberg, Germany; 6 Department of Pediatrics, University of Ulm, Prittwitzstraße 43, 89075 Ulm, Germany; 7 Department of Urology, University of Heidelberg, Im Neuenheimer Feld 110, 69120 Heidelberg, Germany

## Abstract

**Background:**

Chemotherapy for pancreatic carcinoma often has severe side effects that limit its efficacy. The glucocorticoid (GC) dexamethasone (DEX) is frequently used as co-treatment to prevent side effects of chemotherapy such as nausea, for palliative purposes and to treat allergic reactions. While the potent pro-apoptotic properties and the supportive effects of GCs to tumour therapy in lymphoid cells are well studied, the impact of GCs to cytotoxic treatment of pancreatic carcinoma is unknown.

**Methods:**

A prospective study of DEX-mediated resistance was performed using a pancreatic carcinoma xenografted to nude mice, 20 surgical resections and 10 established pancreatic carcinoma cell lines. Anti-apoptotic signaling in response to DEX was examined by Western blot analysis.

**Results:**

*In vitro*, DEX inhibited drug-induced apoptosis and promoted the growth in all of 10 examined malignant cells. *Ex vivo*, DEX used in physiological concentrations significantly prevented the cytotoxic effect of gemcitabine and cisplatin in 18 of 20 freshly isolated cell lines from resected pancreatic tumours. No correlation with age, gender, histology, TNM and induction of therapy resistance by DEX co-treatment could be detected. *In vivo*, DEX totally prevented cytotoxicity of chemotherapy to pancreatic carcinoma cells xenografted to nude mice. Mechanistically, DEX upregulated pro-survival factors and anti-apoptotic genes in established pancreatic carcinoma cells.

**Conclusion:**

These data show that DEX induces therapy resistance in pancreatic carcinoma cells and raise the question whether GC-mediated protection of tumour cells from cancer therapy may be dangerous for patients.

## Background

Pancreatic cancer remains an unfortunate disease with a 5-year survival rate below 1%. Thus, it represents one of the leading causes of cancer related death in industrialized countries despite advances in medical therapy and surgical techniques [1]. One of the major hallmarks of pancreatic cancer is its early systemic dissemination and its extraordinary local tumour progression. These features may contribute to more than 3/4 of patients diagnosed with this disease who cannot be offered curative treatment and, therefore, may determine the high mortality rate among patients with pancreatic cancer [2]. Surgery is the mainstay of treatment. Chemotherapy is important in controlling residual disease following surgery and may be used as neoadjuvant therapy in patients with advanced disease. The standard chemotherapy for advanced pancreatic cancer is currently gemcitabine. Alternatively, combinations of mitomycin C, cisplatin and 5-fluorouracil [3] may be used and are routinely given together with dexamethasone (DEX), a synthetic glucocorticoid (GC).

DEX and similar GCs were first introduced to tumour therapy on the basis of pro-apoptotic effects in lymphoid cells and on their effectiveness in treating tumour related edema, inflammation, pain, and electrolyte imbalance as well as stimulating appetite, and most importantly, preventing nausea and emesis caused by cytotoxic drugs [4-8]. However, controlled randomized trials evaluating a potential impact of GCs on growth of solid tumours and patient survival have never been performed. Concerns about the widespread use of GCs during therapy of solid tumours have been expressed repeatedly [9,10] involving studies showing enhanced percentages of metastases in breast cancer patients [11,12] or an increased risk of skin cancer and non-Hodgkin lymphomas among users of systemic GCs [13]. Recent studies suggest inhibition of cytotoxic therapy-induced apoptosis by DEX in established lung, cervical and breast carcinoma cell lines [14-16]. For inhibition of apoptosis a functional glucocorticoid receptor (GR) is required and association with the transcriptional induction of survival molecules like serum and GC-inducible protein kinase-1 (SGK-1) and mitogen-activated protein kinase phosphatase-1 (MKP-1) is suggested in human established breast cancer cell lines [15]. Thus, treatment with a GC alone or combined with chemotherapy led to an increased protein expression of SGK-1 and MKP-1 while specific inhibition of these molecules by small interfering RNA reversed the anti-apoptotic effects of GC treatment in breast cancer cells [15].

To analyze whether DEX might affect the outcome of cytotoxic therapy of pancreatic carcinomas, cell lines and tumour cells freshly isolated from resected pancreatic cancer specimen as well as a tumour xenograft on nude mice were examined. Cells were treated *in vitro *or *ex vivo *with gemcitabine or cisplatin in the presence or absence of DEX. DEX inhibited apoptosis and promoted proliferation of the cancer cells independently of age, gender, histology or TNM and induced therapy resistance even in an *in vivo *xenograft model. Differential regulation of expression of pro-survival factors and anti-apoptotic genes may be the underlying mechanism for cell-type specific DEX-induced resistance.

## Methods

### Pancreatic carcinoma cell lines and culture

The established pancreatic carcinoma cell lines were derived from the tumour bank of the German Cancer Research Center (Capan-1, Capan-2, DAN-G), or from the lab of H. Friess (SU8686, PANC-1, MIA-PaCa2, BxPc-3, Colo-357, T3M4 and AsPC-1). Cells were grown at 37°C in DMEM. DMEM was obtained from Life Technologies Gibco BRL (Karlsruhe, Germany) supplemented with 10% heat-inactivated fetal bovine serum (Sigma, Deisenhofen, Germany), 25 mM HEPES and 2 mM L-glutamine (all from Gibco/Life Technologies, Paisley, Scotland).

### Isolation of fresh tumour cells from resected pancreatic cancer specimen

Solid tumours fromfreshpancreatic carcinomas were minced in RPMI medium supplemented with 20% heat-inactivated fetal bovine serum (Sigma, Deisenhofen, Germany), 25 mM HEPES, 2 mM L-glutamine and Pen/Strep (all from Gibco/Life Technologies, Paisley, Scotland) under sterile conditions, counted by trypan blue exclusion and immediately analyzed by the MTT-assay. Patient material was obtained under the approval of the ethic committee of the University of Heidelberg. Diagnoses were established by conventional clinical and histological criteria according to the World Health Organization (WHO). All surgical resections were indicated by principles and practice of oncological therapy.

### Nude mice and xenografts

Cells of the pancreatic carcinoma cell line MIA-PaCa2 were injected subcutaneously into the right anterior flank of 6-10 weeks old NMRI (nu/nu) female mice. After the tumours had reached a mean diameter of about 8-10 mm, mice carrying MIA-PaCa2 xenografts were randomized divided into groups of 5-6 animals each and treatment was started. The mice were given 0.28 mg/l DEX in the drinking water, and the daily amount of water consumed was approximately 30 ng/g body weight. A mouse with a body weight of 25 g drinks 2.5 ml (1/10 body weight) per day corresponding to a serum concentration of about 0.07 μM DEX. Two days after adding DEX to the drinking water, therapy with cisplatin was started (day 0). Cisplatin (5 mg/kg) was injected at two consecutive days (day 2 and day 3). In detail, we diluted 10 mg cisplatin in 20 ml PBS (0.5 mg CIS/ml) and injected 0.01 ml of this solution per g mouse. That means, we injected 0.25 ml in a mouse of 25 g body weight. Tumour growth was followed by measuring two diameters daily with calipers and tumour volumes (v) were calculated using the formula v = 1/2 (length × width^2^). Mice were humanely euthanized at tumour sizes > 3000 mm^3^. Animal experiments have been carried out in the animal facilities of the DKFZ after approval by the authorities (Regierungspräsidium Karlsruhe).

### Drugs

A stock solution of cisplatin (Sigma, Deisenhofen, Germany) was prepared in DMSO at the concentration of 33 mM. Gemcitabine (kind gift from Eli Lilly, Indianapolis, IN, USA) was diluted in PBS to a 50 μM stock. A 25 mM stock of DEX (Sigma) was prepared in ethanol. Final concentrations of the solvents in medium were 0.1% or less.

### Measurement of apoptosis

Cells were stained with fluoresceinthiocyanate (FITC)-conjugated annexin V (BD Biosciences, Heidelberg, Germany) and externalization of phosphatidylserine as well as the forward side scatter profile were identified by flow cytometry (FACScan, BD Biosciences) as described [14]. In parallel, DNA-fragmentation was measured by the nicoletti method as described [17] or cell morphology was determined by the FSC/SSC profile.

### MTT-assay

Primary tumour cells were resuspended at 5 × 10^5^/ml in 96-well microplates, 100 μl per well. After treatment, the MTT-assay was performed as recently described [16].

### Western blot analysis

Equal numbers of cells in each experimental condition were lysed with 2 x Laemmli buffer and were fractionated on 10% SDS-PAGE gels. The fractionated proteins were transferred to nitrocellulose and were stained with Ponceau S dye to confirm equal protein loading. The membranes were then rinsed and incubated in the rabbit polyclonal antibodies MKP-1, BAG-1 (both from Santa Cruz, Heidelberg, Germany), SGK-1 (Stressgene, Victoria, Canada), X-IAP (New England Biolabs/Cell Signaling, Frankfurt, Germany) or in the mouse monoclonal antibodies: BCL-2 (Merck/Calbiochem, Darmstadt, Germany), ACTIN (clone C4, ICN, Aurora, Ohio, USA). Bound antibodies were detected by anti-rabbit or anti-mouse/horseradish peroxidase conjugates (Santa Cruz) and enhanced chemiluminescence system.

### Statistical analysis

#### Surgical specimen

each tumour probe was investigated in a 2-factorial design consisting of three doses of DEX (0.1 = DEX1, 1 = DEX2, 10 μM = DEX3) and a control and three doses of gemcitabine (25 nM = 1; 50 nM = 2; 200 nM = 3) or cisplatin (7 μM = 1; 17 μM = 2; 34 μM = 3) and a control (= 0). This results in a total of 16 experimental conditions. Viability of the cells under each condition was determined as mean of 3 to 8 replicates together with its standard deviation and then standardized on the result of the double control (no cytotoxic agent and no DEX applied); i.e. the viabilities of all conditions were divided by the mean of the double control. For each tumour probe the standardized means were compared separately for each therapeutic dose and for the control by comparing the three DEX doses with its respective control (cisplatin or gemcitabine treatment alone). Notice, that the four means (three DEX doses and control) under the condition of no therapy describe the effect of DEX alone while the other three sets of four means under the three doses of the therapeutic agent describe the resistance of the cells under treatment depending on DEX. We declared a DEX dose group resistant when its mean minus one standard deviation (X¯j
 MathType@MTEF@5@5@+=feaafiart1ev1aaatCvAUfKttLearuWrP9MDH5MBPbIqV92AaeXatLxBI9gBaebbnrfifHhDYfgasaacH8akY=wiFfYdH8Gipec8Eeeu0xXdbba9frFj0=OqFfea0dXdd9vqai=hGuQ8kuc9pgc9s8qqaq=dirpe0xb9q8qiLsFr0=vr0=vr0dc8meaabaqaciaacaGaaeqabaqabeGadaaakeaacuWGybawgaqeamaaBaaaleaacqWGQbGAaeqaaaaa@2F86@ - *SD_j_*) was still larger than the mean plus one standard deviation (X¯0
 MathType@MTEF@5@5@+=feaafiart1ev1aaatCvAUfKttLearuWrP9MDH5MBPbIqV92AaeXatLxBI9gBaebbnrfifHhDYfgasaacH8akY=wiFfYdH8Gipec8Eeeu0xXdbba9frFj0=OqFfea0dXdd9vqai=hGuQ8kuc9pgc9s8qqaq=dirpe0xb9q8qiLsFr0=vr0=vr0dc8meaabaqaciaacaGaaeqabaqabeGadaaakeaacuWGybawgaqeamaaBaaaleaacqaIWaamaeqaaaaa@2F17@ + *SD_0_*) of the respective control group of that tumour probe, j = 1, 2, 3 denoting the three dose groups receiving cytotoxic drugs. On this basis, scores were calculated per therapeutic dose (values 0, 1, 2, 3) as well as per tumour probe in total (values ranging from 0 to 9). A tumour probe was declared as being significantly resistant to cytotoxic treatment when it showed a score of 2 for at least one drug dose or when it reached the total score of 5 out of a maximum of 9 per time point (i.e. more than 50%). A calculation is given as example in Table 1 for tumour probe 2.

Please note, that the scores and definition of resistance have been exclusively designed for the present study by L. Edler and W. Rittgen from the Department of Biostatistics, German Cancer Research Center.

The outcomes of a group of tumour probes were summarized in respective ratios obtained by adding the individual scores for each condition as well as in total over the number of probes and by dividing through the number of probes (compare Table 1). The results of the evaluation of each single tumour probe are available upon request. The tumour population was considered as suffering from resistance as a whole, when for one therapeutic dose all ratios were higher than 50% or when 5 of the total of 9 combinations were higher than 50%. The statistical method described was applied independently to all three time points.

#### Established cell lines

Corresponding to the analysis of patient-derived tumour probes, cell lines were investigated in a 2-factorial design consisting of one dose of DEX (1 μM) and a control and three doses of cytotoxic treatment and a control resulting in a total of 8 experimental conditions. Viability of the cells under each condition was determined as mean of 8 replicates (MTT-assay) or as mean of 6 replicates (apoptosis) as described above.

#### Xenografts on nude mice

A distribution free test for tumour growth curve analyses was used for therapy experiments with xenografted cancer cells as described [18].

## Results

### DEX induces resistance towards gemcitabine and cisplatin *in vitro*

**Figure 1:**
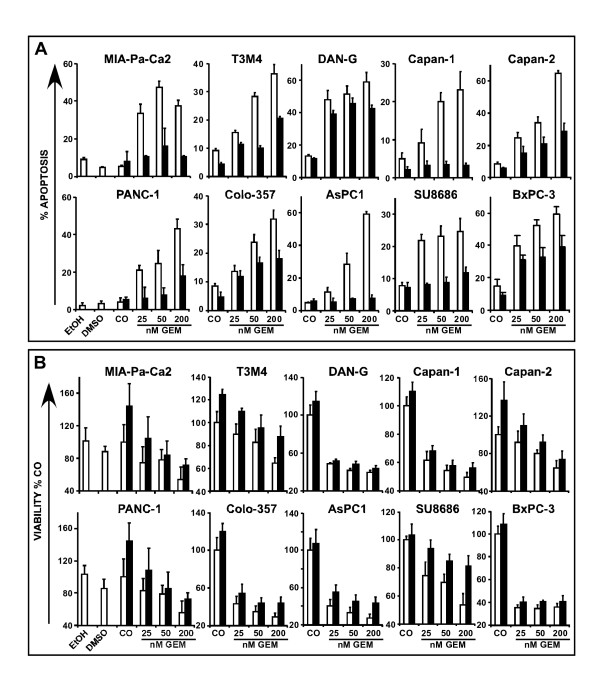
**DEX inhibits apoptosis and promotes proliferation in response to gemcitabine *in vitro*. **The established pancreatic cancer cells MIA-Pa-Ca2, T3M4, DAN-G, Capan-1, Capan-2, PANC-1, Colo-357, AsPC1, SU8686 and BxPc-3 were left either untreated (CO) or were treated with gemcitabine (GEM: 25, 50, 200 μM) in the absence (white bars) or presence (black bars) of DEX (1 μM) which was added 48 h prior to cytotoxic treatment. 72 h following addition of gemcitabine, (A) apoptosis was analyzed by staining of the cells with annexin-FITC and FACS-analysis. (B) Likewise, viability was detected by the MTT-assay. Experiments were performed three times with identical outcome and standard deviations are shown.

**Figure 3:**
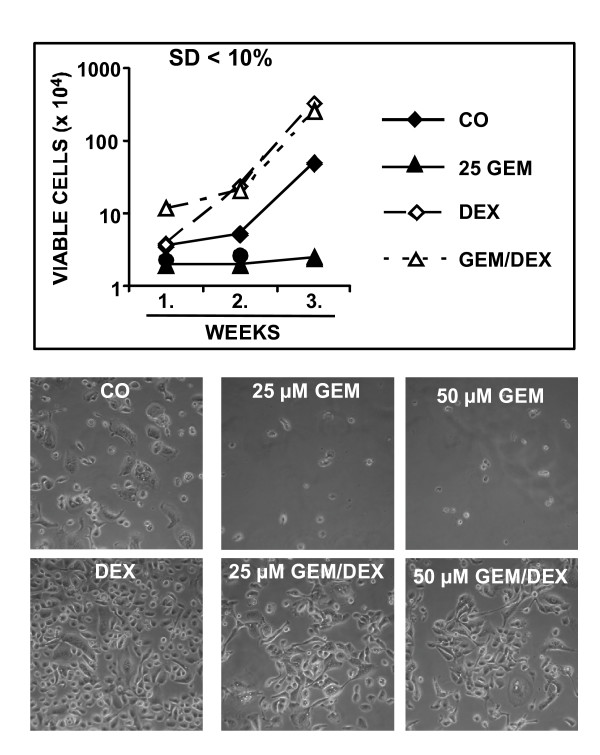
**DEX-pretreated cells are protected despite three weeks incubation with gemcitabine *in vitro*. **AsPC1 cells were left untreated (CO) or were treated with 0.1 μM DEX (DEX), 25 μM GEM (GEM), or both together (DEX/GEM). One, two and three weeks later the amount of viable cells was determined by trypan blue exclusion and cells were photographed after two weeks of incubation. Experiments were performed three times with identical outcome.

To investigate whether DEX might protect pancreatic carcinoma cell lines by interfering with apoptosis we treated MIA-Pa-Ca2, T3M4, DAN-G, Capan-1, Capan-2, PANC-1, Colo-357, AsPC1, SU8686 and BxPc-3 cells with gemcitabine or cisplatin in the presence or absence of DEX. 72 h later, apoptosis was detected by annexin-FITC followed by FACS-analysis. While cytotoxic drugs alone strongly induced apoptosis, the presence of DEX inhibited this effect in all cell lines examined (Fig. 1A, 2A). No induction of apoptosis occurred in untreated cells or cells treated with the solvents alone. These data are confirmed by the measurement of DNA-fragmentation by the nicoletti method or by determining the cell morphology by the FSC/SSC profile and FACS-analysis " [see Additional file I]". In light of inhibited apoptosis we next analyzed whether DEX might influence growth of pancreatic carcinoma cell lines treated with cytotoxic drugs. The same set of cells was treated as described above and viability was detected by the MTT-assay. While gemcitabine or cisplatin alone strongly reduced viability, the presence of DEX diminished the cytotoxic effect in all cell lines (Fig. 1B, 2B). In order to know whether this protective effect of DEX might be long lasting we treated pancreatic cancer cells with DEX in the presence or absence of gemcitabine. One, two and three weeks later viable cells were counted by trypan blue exclusion and cells were photographed after two weeks (Fig. 3). DEX strongly enhanced basal proliferation and increased the viability of gemcitabine-treated cells above levels of untreated controls. Thus, DEX inhibits apoptosis and promotes proliferation of pancreatic carcinoma cells after treatment with cytotoxic drugs *in vitro*.

### DEX induces resistance towards gemcitabine and cisplatin *ex vivo*

**Figure 4:**
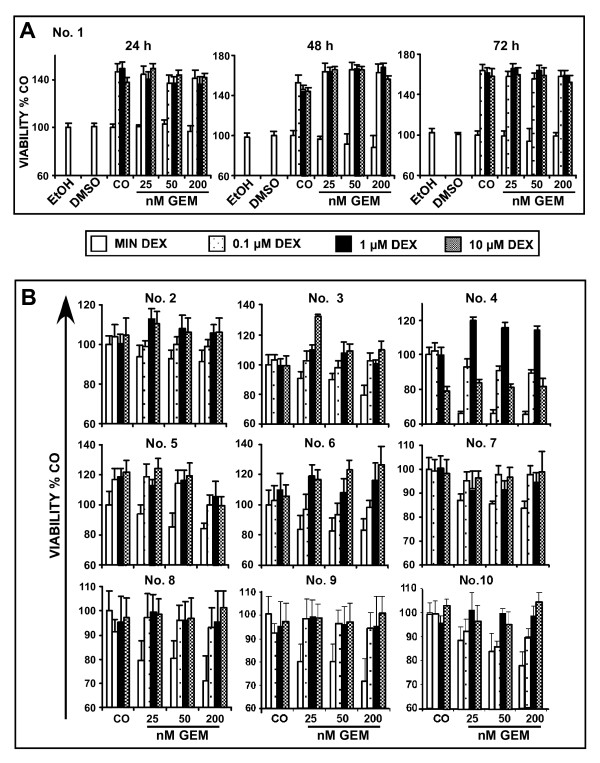
**DEX inhibits the therapeutic effect of gemcitabine *ex vivo*. **(A) Tumour cells from a patient (No. 1) with pancreatic cancer were freshly isolated and cultivated in a concentration of 5 × 10^5^/ml in the absence (white bars) or presence of DEX (0.1, 1 or 10 μM as indicated) for 24 h. Gemcitabine was added (25, 50, 200 nM GEM) while the controls remained either untreated (CO) or were treated with the solvents alone (EtOH, used for dilution of DEX and DMSO, used for dilution of gemcitabine and cisplatin). 48 h after adding gemcitabine or solvents alone, viability was measured by the MTT-assay. (B) Tumour cells from patients with pancreatic cancer (No. 2-10) were freshly isolated, cultivated, and analyzed as described above. Eight wells per treatment were analyzed and standard deviations are less than 10%. Statistical analysis of significance of DEX-induced resistance is shown in Table III.

**Figure 5:**
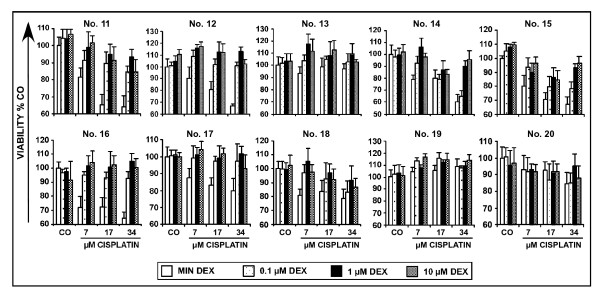
**DEX inhibits the therapeutic effect of cisplatin *ex vivo*. **(A) Tumour cells from patients (No. 11-20) with pancreatic cancer were isolated, cultivated and analyzed as described in Fig. 4, except of that cisplatin (7, 17 34 μM) was used instead of gemcitabine.

To examine the *ex vivo *effect of DEX we isolated tumour cells from pancreatic cancer specimen immediately after resection. The pancreatic tissue used contained 90% pancreatic tumour cells morphologically identified by a pathologist. DEX was used in concentrations of 0.1, 1 and 10 μM from which the median concentration resembles peak plasma levels in the clinical setting [6,19,20]. Cells derived from 20 patients were treated with three concentrations each of gemcitabine or cisplatin in the presence or absence of low, median and high concentrations of DEX. 24, 48 and 72 h later viability was measured (Fig. 4, 5) and [see Additional Files II, IIIIV, V]". While cytotoxic drugs alone strongly reduced viability in all cells, viability was significantly enhanced in 18 of 20 examined primary cell lines. Using statistical analysis viability was significantly enhanced in 50% of cells treated with 0.1 μM DEX, in 71% of cells treated with 1 μM DEX and in 74% of cells treated with 10 μM DEX as calculated from the means of data obtained with three concentrations of cytotoxic therapy at 24, 48 and 72 h after treatment (Table 2, 3). Even DEX alone significantly enhanced basal viability at the low concentration in 9%, at the median concentration in 22% and at the high concentration in 14% of cells while solvents alone had no effect (Fig. 4A and Table 2). Induction of therapy resistance by DEX did neither correlate with the histological phenotype nor with the tumour stage, age or gender of patients (Table 4).

### DEX induces therapy resistance *in vivo*

**Figure 6:**
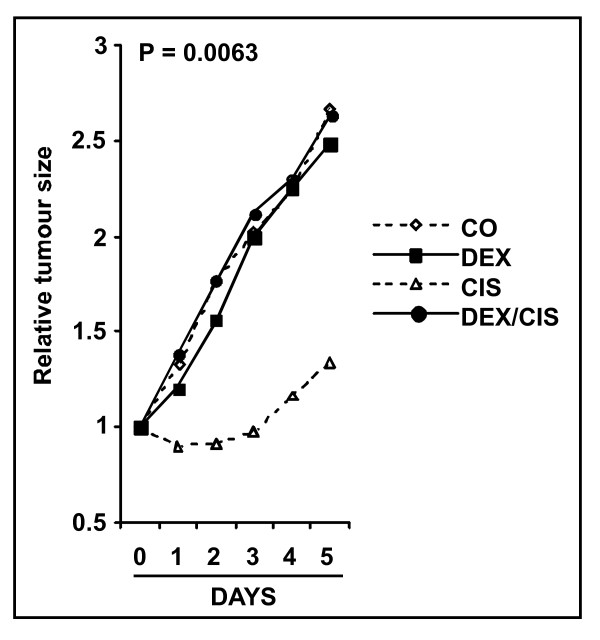
**DEX inhibits therapy-induced carcinoma regression *in vivo*. **MIA-Pa-Ca2 tumour cells were established as xenograft cell line and injected subcutaneously into nude mice treated as described in material and methods. The volumes of the fast-growing tumours are shown from day one to five after start of cisplatin therapy. Data are presented as mean of 5-6 animals and the single measurements are indicated. Statistical analysis: The P-value of 0.0063 for the CO versus CIS versus DEX versus CIS/DEX was determined according to the Koziol test for tumour growth curve analyses [18].

To analyze the *in vivo *effect of DEX we xenografted MIA-PaCa2 cells to nude mice. Tumour volumes are shown at day one to five after start of cisplatin therapy and found to be reduced in mice treated with cisplatin alone (Fig. 6). However, DEX totally prevented the growth-inhibiting effect of cisplatin since the tumours grew as fast as those of untreated control mice.

### MKP-1, SGK-1, X-IAP and Bcl-2 but not BAG-1 may be involved in DEX-induced resistance

**Figure 7:**
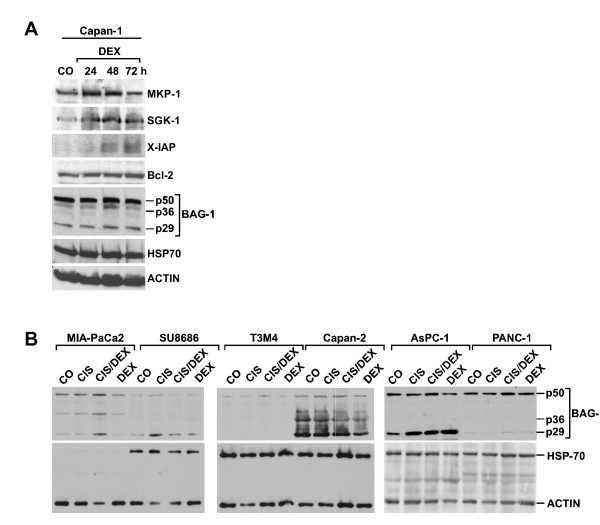
**Upregulation of survival- and anti-apoptotic molecules by DEX.**(A) Capan-1 cells were treated with DEX (1 μM) for 24, 48 or 72 h as indicated. Cells were lysed, proteins were harvested and analyzed by Western blot analysis. (B) MIA-Pa-Ca2, SU8686, T3M4, Capan-2, AsPC1 and PANC-1 were treated as described in Fig. 1. 48 h later proteins were harvested. Western blot analysis using antibodies toward BAG-1 and HSP-70 were performed. The p50, p36 and p29 subunits of BAG-1 as well as the band of HSP-70 are indicated. ACTIN expression is a marker for equal conditions. Antibodies used are: MKP-1 (42 kD), SGK-1 (50-55 kD), X-IAP (57 kD), Bcl-2 (28 kD), BAG-1 (p50, p36 and p29 subunits) HSP-70 (70 kD) and ACTIN (44 kD). The experiments have been performed three times with similar outcome.

In a recent study we demonstrated that DEX-co-treatment induces apoptosis resistance in an established lung and cervical carcinoma cell line but not in leukemic T cells. This was due to differential regulation of diverse apoptosis genes and depends on a functional GR [14]. The upstream mechanism by which DEX inhibits apoptosis and promotes proliferation in pancreatic carcinomas is totally unknown. Therefore, we analyzed the expression of BAG-1, known to link GR-signaling to impaired apoptosis and enhanced proliferation by interaction with HSP-70 [21] by Western blot analysis. While the p50 subunit of BAG-1 could be detected in all cell lines examined, the p36 subunit was only present in MIA-Pa-Ca2, Capan-1 and Capan-2 cells, while the p29 subunit was only present in MIA-Pa-Ca2, SU8686, Capan-1, Capan-2, ASPC-1 and weakly in PANC-1 cells (Fig. 7A, 7B). Induction of either subunit by DEX alone or in combination with cisplatin did not occur. Also, HSP-70, the co-worker of BAG-1, is equally and strongly expressed under any condition in all cell lines except MIA-Pa-Ca2 expressing only minimal amounts of HSP-70. As a control, we examined expression of GR, and found the protein product whose level was also not changed in response to cisplatin and/or DEX (data not shown). Since enhanced expression of MKP-1 and SGK-1 has been recently described to be involved in DEX-induced resistance in established breast cancer cell lines [15], we examined expression of these pro-survival molecules in pancreatic cancer cells by Western blot analysis together with expression of the anti-apoptotic molecules X-IAP and Bcl-2. During a time kinetic from 24 to 72 h, DEX strongly enhanced the levels of MKP-1, SGK1 and X-IAP, in several pancreatic cancer cell lines examined, while Bcl-2 expression was only marginally increased. Representative data with Capan-1 cells are shown (Fig. 7A). Therefore, enhanced expression of MKP-1, SGK-1, X-IAP and Bcl-2 but not of BAG-1 may be involved in DEX-induced therapy resistance of pancreatic carcinomas.

## Discussion

Our data demonstrate for the first time that *in vitro*, *ex vivo *and *in vivo *treatment of pancreatic cancer cells with GCs induces resistance towards cytotoxic therapy. We report here, that DEX inhibits gemcitabine- and cisplatin-induced apoptosis and promotes proliferation in all of ten examined established pancreatic cancer cell lines, in 18 of 20 primary pancreatic tumour cell lines isolated from cancer specimen and in xenografted pancreatic tumour cells. These results suggest a cell type specific effect of GCs, since these agents are well known to act pro-apoptotic and anti-proliferative in lymphoid cells [10,22]. The observed induction of therapy resistance by DEX in pancreatic carcinoma did neither correlate with the histological phenotype nor with the tumour stage, age or gender of patients corresponding to our recent results obtained with DEX-treated malignant cells from lung [16], prostate, kidney, bladder, testis [23], colon, rectum, liver [24] and ovary [25]. Thus, our results obtained with pancreatic carcinomas may be transferred to other solid tumours, since we found induction of resistance by GCs in 95% of more than 160 examined fresh surgical specimen, xenografts on mice and established cell lines of tumours including bladder, bone, brain, breast, cervix, colon, liver, lung, kidney, ovary, prostate, rectum and testis, together with neuroblastomas, and melanomas (compare our recent manuscripts [16,23-25]and our unpublished work). Thus, the present findings strongly suggest that GCs are highly suspicious to induce resistance to cytotoxic therapy in clinical settings, specifically, in patients with pancreatic cancer. This point was recently addressed in a retrospective clinical study evaluating records of 245 of a total of 763 patients with ovarian carcinoma and no negative outcome of GC treatment on survival of patients was found [26]. However, this study cannot give a definitive answer to the question of whether GC treatment, given as part of anti-emetic regimen, prevention of allergic reactions or as immunosuppressive therapy is safe in patients with ovarian carcinoma or other solid tumours – the reasons are discussed elsewhere [27]. Furthermore, there are other clinical examinations which clearly show a negative impact of GCs, e.g. an increased metastatic potential in breast cancer patients and an enhanced risk of skin cancer and lymphomas among users of systemic GCs [11-13].

The mechanisms by which GCs induce apoptosis in lymphoid cells are well studied. These include depolarization of the mitochondrial membrane potential, enhanced expression of the death receptor CD95 and its ligand, followed by activation of the caspase cascade [28-30]. The same mechanisms that are induced in lymphoid cells are blocked in several carcinoma cells by GCs thereby inhibiting chemo- and radiation therapy-induced apoptosis [14,15].An open question is, how GCs mediate these cell-type specific effects clearly shown to be related to a functional glucocorticoid receptor (GR) [14,15]. Nothing is known about a direct link between GR signaling and induction of apoptosis. One candidate molecule is the anti-apoptotic co-chaperone BAG-1 which together with the chaperone HSP-70 is involved in regulation of GR binding activity [21]. However, DEX did not up-regulate expression of BAG-1 in our studies, neither basal, nor in the presence of cisplatin, as would have been expected in the case of an anti-apoptotic influence of BAG-1 on GR signaling. In contrast, we found DEX-induced upregulation of the pro-survival genes SGK-1 and MKP-1. This is in line with another report demonstrating that downregulation of MKP-1 in both pancreatic cancer and chronic pancreatitis suppresses tumourigenicity of pancreatic cancer cells [31]. Similarly, Bcl-2 overexpression has been associated with acquired resistance of pancreatic carcinoma [32] corresponding to our data which show upregulation X-IAP and to a somehow weaker extent of Bcl-2 in response to DEX. Therefore, cell type-specific anti-apoptotic and pro-proliferative signaling may be involved in induction of resistance by GCs in pancreatic carcinoma cells.

Another possible explanation for the cell-type specific anti-apoptotic properties of GCs may be the differential expression of GR co-activators and co-repressors in diverse cell types, as proposed to explain the opposite effects of tamoxifen on mammary versus endometrial tissue [33]. A recent study compared gene expression of a breast cancer cell line with genes found to be regulated by DEX in lymphocytes [15]. Surprisingly, only a few of the genes regulated by DEX in carcinomas are the same as those identified as GC-regulated in lymphocytes. Among the differential regulated set of sequences are apoptotic genes as well as genes involved in signal transduction, metabolism, transcription, cell cycle, DNA repair and others. These recent data strongly suggest that tissue-specific differences in GC-induced apoptosis versus survival outcomes may be due to cell-type-specific transcriptional regulation.

## Conclusion

In conclusion, we show by *in vitro*,*ex vivo *and *in vivo *studies that application of DEX renders pancreatic cancer cells resistant to apoptosis and promotes proliferation following cytotoxic therapy. This is in contrast to the effect of GCs in lymphoid cells and may involve cell type specific regulation of survival molecules such as SGK-1 and MKP-1 together with anti-apoptotic molecules such as X-IAP and Bcl-2. Thus, while some properties of GCs may be of benefit, induction of resistance in tumour cells towards cancer therapy may be dangerous for patients. Prospective clinical studies which do not exist until now, are urgently needed.

## Abbreviations

Dexamethasone (DEX), Glucocorticoids (GCs), Glucocorticoid Receptor (GR)

## Competing interests

The author(s) declare that they have no competing interests.

## Authors' contributions

CZ: performed the experiments with primary and established cell lines

AK: selected tumors and participated in creating the studies

PB: has been involved in drafting the manuscript for important intellectual content

ACBC: has been intellectually involved in studying mechanisms of DEX-induced resistance and in preparation of the manuscript

JM: performed the xenograft studies

WR: performed statistical analyses

LE: performed statistical analyses

KMD: was involved in drafting the manuscript critically for important intellectual content

MB: organized transfer of freshly resected tumors

HF: organized transfer of freshly resected tumors and participated in design of the studies and preparation of the manuscript

IH: concepted and designed the study, analysed and interpretated the data and wrote the manuscript

All authors read and approved the final manuscript

## Pre-publication history

The pre-publication history for this paper can be accessed here:



## Supplementary Material

Additional File I **DEX inhibits apoptosis in response to gemcitabine *in vitro ***The established pancreatic cancer cells T3M4, Colo-357 and Capan-2 were left either untreated (CO) or were treated with gemcitabine (GEM: 25, 50, 200 μM) in the absence (white bars) or presence (black bars) of DEX (1 μM) which was added 48 h prior to cytotoxic treatment. 72 h following addition of gemcitabine, (A) apoptosis was analyzed by staining of the cells with annexin-FITC (% APOPTOSIS), with nicoletti buffer (% DNA-FRAGMENTATION) and FACS-analysis or cells were left unstained and cell morphology was analyzed by FACS analysis (% CELL DEATH). Click here for file

Additional File II **DEX induces resistance *ex vivo ***Tumour cells from 20 patients (No. 1-20) with pancreatic cancer were freshly isolated and cultivated in a concentration of 5 × 10^5^/ml in the absence (white bars) or presence of DEX (0.1, 1 or 10 μM as indicated) for 24 h. Gemcitabine or cisplatin were added in concentrations indicated while the controls remained untreated (CO) or were treated with the solvents alone. 24, 48 and 72 h after adding cytotoxic drugs, viability was measured by the MTT-assay. Eight wells per treatment were analyzed and standard deviations are less than 10%. Click here for file

Additional File III **DEX induces resistance *ex vivo ***Tumour cells from 20 patients (No. 1-20) with pancreatic cancer were freshly isolated and cultivated in a concentration of 5 × 10^5^/ml in the absence (white bars) or presence of DEX (0.1, 1 or 10 μM as indicated) for 24 h. Gemcitabine or cisplatin were added in concentrations indicated while the controls remained untreated (CO) or were treated with the solvents alone. 24, 48 and 72 h after adding cytotoxic drugs, viability was measured by the MTT-assay. Eight wells per treatment were analyzed and standard deviations are less than 10%. Click here for file

Additional File IV **DEX induces resistance *ex vivo ***Tumour cells from 20 patients (No. 1-20) with pancreatic cancer were freshly isolated and cultivated in a concentration of 5 × 10^5^/ml in the absence (white bars) or presence of DEX (0.1, 1 or 10 μM as indicated) for 24 h. Gemcitabine or cisplatin were added in concentrations indicated while the controls remained untreated (CO) or were treated with the solvents alone. 24, 48 and 72 h after adding cytotoxic drugs, viability was measured by the MTT-assay. Eight wells per treatment were analyzed and standard deviations are less than 10%. Click here for file

Additional File V **DEX induces resistance *ex vivo ***Tumour cells from 20 patients (No. 1-20) with pancreatic cancer were freshly isolated and cultivated in a concentration of 5 × 10^5^/ml in the absence (white bars) or presence of DEX (0.1, 1 or 10 μM as indicated) for 24 h. Gemcitabine or cisplatin were added in concentrations indicated while the controls remained untreated (CO) or were treated with the solvents alone. 24, 48 and 72 h after adding cytotoxic drugs, viability was measured by the MTT-assay. Eight wells per treatment were analyzed and standard deviations are less than 10%. Click here for file

## Figures and Tables

**Figure 2:**
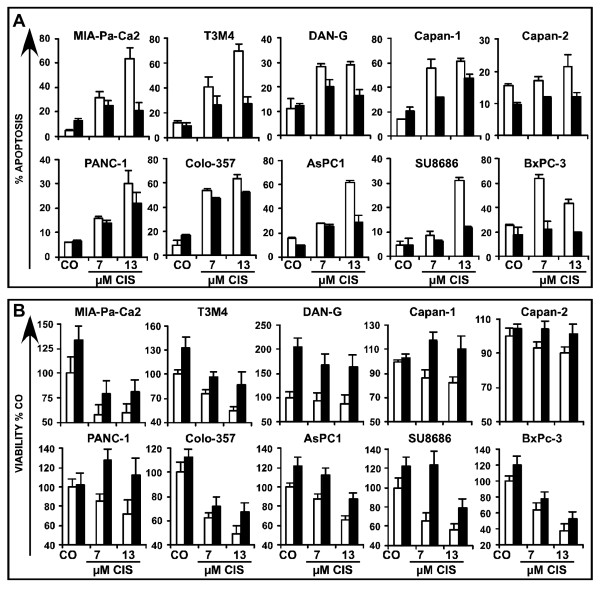
**DEX inhibits apoptosis and promotes proliferation in response to cisplatin *in vitro*. **Cells were treated and analyzed as described in Fig. 1 except that cisplatin (7, 13 μM) was used instead of gemcitabine.

**Table 1:** Calculation of resistance for tumour probe 2.

**Time**	**Drug**	**Treatments**	**DEX1**	**DEX2**	**DEX3**	**Sum**
24 h	0	8/8/8/8	0	0	0	**0**
	
	1	8/8/5/5	0	1	1	**2**
	2	7/8/4/8	0	1	1	**2**
	3	7/8/8/7	0	1	1	**2**

48 h	0	8/8/8/8	0	0	0	**0**
	
	1	8/8/8/8	0	1	1	**2**
	2	8/8/8/8	0	1	1	**2**
	3	8/8/8/8	0	1	1	**2**

72 h	0	7/8/6/7	0	1	0	**1**
	
	1	3/7/6/4	0	0	1	**1**
	2	7/8/6/8	0	1	1	**2**
	3	8/5/8/8	0	1	1	**2**

Time: Cells were treated with chemotherapy for 24, 48 or 72 h. Drug: Cells were left either untreated (0) or were treated with three different concentrations of chemotherapy (1, 2, 3). Treatments: the number of repeated measurements from which means and standard deviations were calculated are indicated. DEX1, DEX2, DEX3: Non-drug treated cells (Drug 0) or drug-treated cells (Drug 1, 2, 3) were treated with three different concentrations of DEX. The value of 0 or 1 was assigned depending on whether or not the DEX group was declared resistant. There is a maximum score of 9 per time point for drug-treated cells. The basal effect of DEX (drug 0 row) was evaluated for its own and is not included in the sum column.

**Table 2:** DEX-induced resistance in fresh patient-derived pancreatic tissue.

**Time**	**No. of patients**	**Drugs**	**DEX1**	**DEX2**	**DEX3**	**Sum**
24 h	18	0	11%	22%	11%	**15**%
		1	44%	78%	83%	**69**%
		2	44%	67%	56%	**56**%
		3	56%	72%	90%	**72**%

48 h	20	0	10%	15%	15%	**13**%
		1	60%	70%	90%	**73**%
		2	50%	75%	65%	**63**%
		3	60%	75%	70%	**68**%

72 h	18	0	56%	28%	17%	**17**%
		1	44%	72%	67%	**61**%
		2	39%	67%	67%	**57v**
		3	56%	67%	78%	**67**%
**Sum of drug-treated cells 1-3**			**50**%	**71**%	**74**%	
	
**Sum of control cells 0**			**9**%	**22**%	**14**%	

The percentage of cells derived from 20 patients which show statistically significant chemotherapy resistance/enhanced viability in response to treatment with dexamethasone is given. For definition of resistance, please see the Material and Methods section. Time: time point after incubation with drugs; No. of patients: indicates the number of patients whose cells were freshly isolated from resected pancreatic tissue (compare Table 2); 0: without drugs, 1: low drug concentration, 2: medium drug concentration, 3: high drug concentration of gemcitabine (25, 50 200 nM) or cisplatin (7,17,34 μM). DEX1: 0.1 μM, DEX2: 1 μM, DEX3: 10 μM.

**Table 3:** Statistical significance of DEX-induced resistance in patients.

Patient No.	24 h	48 h	72 h
1	S	S	S
2	S	S	S
3	S	S	S
4	S	S	S
5	S	S	S
6	S	S	S
7	NS	S	NS
8	S	S	NS
9	S	NS	NS
10	NS	NS	S
11	S	S	S
12	S	S	S
13	S	NS	S
14	S	S	S
15	ND	S	ND
16	ND	S	ND
17	S	S	S
18	S	NS	NS
19	NS	NS	NS
20	NS	NS	NS

Significance of DEX-induced resistance (compare Fig. 4, 5 and Fig. II-V) in patient-derived cells was analysed as described in the Material and Methods section.

S = significant

NS = non-significant

ND = not done

**Table 4:** Characterization of patient-derived fresh pancreatic tissue.

*No*.	*Gender*	*Age (years)*	*Histological Typing (WHO)*	*pTNM*
1	M	84	adeno	pT3, pN1
2	F	55	adenoma	
3	F	80	adeno	pT3, pN1
4	F	56	adeno	pT3, pN0
5	M	44	adeno	pT3, pN1
6	F	65	adeno	pT3, pN1
7	M	75	adono	pT3, pN1
8	M	72	adeno	PT3, pN1
9	F	58	adeno	pT3, pN1
10	M	74	adeno	pT3, pN1
11	F	55	adeno	pT3, pN1
12	F	38	adeno	pT3, pN1
13	M	71	serous adenoma	
14	F	37	acinus cell carcinoma	pT3, pN1
15	M	67	adeno	pT3, pN1
16	F	58	adeno	pT3, pN1
17	F	73	adeno	pT3, pN1
18	M	66	adeno	pT3, pN0
19	M	67	neuroendocrine	
20	M	70	adeno	pT3, pN1

M = male, F = female, No. = number of patient

## References

[B1] Neoptolemos JP, Dunn JA, Stocken DD, Almond J, Link K, Beger H, Bassi C, Falconi M, Pederzoli P, Dervenis C, Fernandez-Cruz L, Lacaine F, Pap A, Spooner D, Kerr DJ, Friess H, Buchler MW (2001). Adjuvant chemoradiotherapy and chemotherapy in resectable pancreatic cancer: a randomised controlled trial. Lancet.

[B2] Keleg S, Buchler P, Ludwig R, Buchler MW, Friess H (2003). Invasion and metastasis in pancreatic cancer. Mol Cancer.

[B3] Petty RD, Nicolson MC, Skaria S, Sinclair TS, Samuel LM, Koruth M (2003). A phase II study of mitomycin C, cisplatin and protracted infusional 5-fluorouracil in advanced pancreatic carcinoma: efficacy and low toxicity. Ann Oncol.

[B4] Cheng L, Du C, Murray D, Tong X, Zhang YA, Chen BP, Hawley RG (1997). A GFP reporter system to assess gene transfer and expression in human hematopoietic progenitor cells. Gene Ther.

[B5] Aapro MS (1991). Present role of corticosteroids as antiemetics.. Recent results in cancer research.

[B6] (1995). The Italian Group for Antiemetic Research. Dexamethasone, granisetron, or both for the prevention of nausea and vomiting during chemotherapy for cancer.. N Engl J Med.

[B7] (2000). The Italian Group for Antiemetic Research. Dexamethasone alone or in combination with ondansetron for the prevention of delayed nausea and vomiting induced by chemotherapy.. N Engl J Med.

[B8] Kirkbride P, Bezjak A, Pater J, Zee B, Palmer MJ, Wong R, Cross P, Gulavita S, Blood P, Sun A, Dundas G, Ganguly PK, Lim J, Chowdhury AD, Kumar SE, Dar AR (2000). Dexamethasone for the prophylaxis of radiation-induced emesis: a National Cancer Institute of Canada Clinical Trials Group phase III study. J Clin Oncol.

[B9] Haid M (1981). Steroid antiemesis may be harmful.. N Engl J Med.

[B10] Rutz HP (2002). Effects of corticosteroid use on treatment of solid tumours. Lancet.

[B11] Iversen HG, Hjort GH (1958). The influence of corticoid steroids on the frequency of spleen metastases in patients with breast cancer. Acta Pathol Microbiol Scand.

[B12] Sherlock P, Hartmann WH (1962). Adrenal  steroids and the pattern of metastases of breast cancer. JAMA.

[B13] Sorensen HT, Mellemkjaer L, Nielsen GL, Baron JA, Olsen JH, Karagas MR (2004). Skin cancers and non-hodgkin lymphoma among users of systemic glucocorticoids: a population-based cohort study. J Natl Cancer Inst.

[B14] Herr I, Ucur E, Herzer K, Okouoyo S, Ridder R, Krammer PH, von Knebel Doeberitz M, Debatin KM (2003). Glucocorticoid co-treatment induces apoptosis resistance toward cancer therapy in carcinomas. Cancer Res.

[B15] Wu W, Chaudhuri S, Brickley DR, Pang D, Karrison T, Conzen SD (2004). Microarray analysis reveals glucocorticoid-regulated survival genes that are associated with inhibition of apoptosis in breast epithelial cells. Cancer Res.

[B16] Gassler N, Zhang C, Schnabel PA, Dienemann H, Debatin KM, Mattern J, Herr I (2005). Dexamethasone-induced cisplatin and gemcitabine resistance in lung carcinoma samples treated ex vivo. Brit J Cancer.

[B17] Nicoletti I, Migliorati G, Pagliacci MC, Grignani F, Riccard C (1991). A rapid and simple method for measuring thymocyte apoptosis by propidium iodide staining and flow cytometry. J Immunol Methods.

[B18] Koziol JA, Maxwell DA, Fukushima M, Colmerauer ME, Pilch YH (1981). A distribution-free test for tumor growth curve analyses with application to an animal tumor immunotherapy experiment. Biometrics.

[B19] Ioannidis JP, Hesketh PJ, Lau J (2000). Contribution of dexamethasone to control of chemotherapy-induced nausea and vomiting: a meta-analysis of randomized evidence. J Clin Oncol.

[B20] Brady ME, Sartiano GP, Rosenblum SL, Zaglama NE, Bauguess CT (1987). The pharmacokinetics of single high doses of dexamethasone in cancer patients. Eur J Clin Pharmacol.

[B21] Cato AC, Mink S (2001). BAG-1 family of cochaperones in the modulation of nuclear receptor action. J Steroid Biochem Mol Biol.

[B22] Rutz HP, Herr I (2004). Interference of glucocorticoids with apoptosis signaling and host-tumor interactions. Cancer Biol Ther.

[B23] Zhang C, Mattern J, Haferkamp A, Pfitzenmaier J, Debatin KM, Hohenfellner M, Rittgen W, Edler L, Groene E, Herr I (2006). Corticosteroid-induced chemotherapy resistance in urological cancers. Cancer Biol Ther.

[B24] Zhang C, Kolb A, Gassler N, Wenger T, Herzer K, Debatin KM, Buechler M, Edler L, Rittgen W, Friess H, Herr I (2005). Dexamethasone desensitizes hepatocellular and colorectal tumours toward cytotoxic therapy. Cancer Letters.

[B25] Zhang C, Marme A, Wenger T, Gutwein P, Edler L, Rittgen W, Debatin KM, Altevogt P, Herr I (2006). Glucocorticoid-mediated inhibition of chemotherapy in ovarian carcinomas. Int J Oncol.

[B26] Muenstedt K, Borces D, Bohlmann MK, Zygmunt M, von Georgi R (2004). Glucocorticoid administration in antiemetic therapy. Cancer.

[B27] Rutz HP, Herr I (2005). Glucocorticoid administration in antiemetic therapy: is it safe?. Cancer.

[B28] Kofler R (2000). The molecular basis of glucocorticoid-induced apoptosis of lymphoblastic leukemia cells. Histochem Cell Biol.

[B29] Planey SL, Litwack G (2000). Glucocorticoid-induced apoptosis in lymphocytes. Biochem Biophys Res Commun.

[B30] Distelhorst CW (2002). Recent insights into the mechanism of glucocorticosteroid-induced apoptosis. Cell Death Differ.

[B31] Liao Q, Guo J, Kleeff J, Zimmermann A, Buchler MW, Korc M, Friess H (2003). Down-regulation of the dual-specificity phosphatase MKP-1 suppresses tumorigenicity of pancreatic cancer cells. Gastroenterology.

[B32] Shi X, Liu S, Kleeff J, Friess H, Buchler MW (2002). Acquired resistance of pancreatic cancer cells towards 5-Fluorouracil and gemcitabine is associated with altered expression of apoptosis-regulating genes. Oncology.

[B33] Shang Y, Brown M (2002). Molecular determinants for the tissue specificity of SERMs. Science.

