# Supplementary figures and images for: Corticosteroid co-treatment induces resistance to chemotherapy in surgical resections, xenografts and established cell lines of pancreatic cancer
Source: BMC Cancer. 2006 Mar 15;6:61. doi: 10.1186/1471-2407-6-61 (PMC1434760; doi:10.1186/1471-2407-6-61)

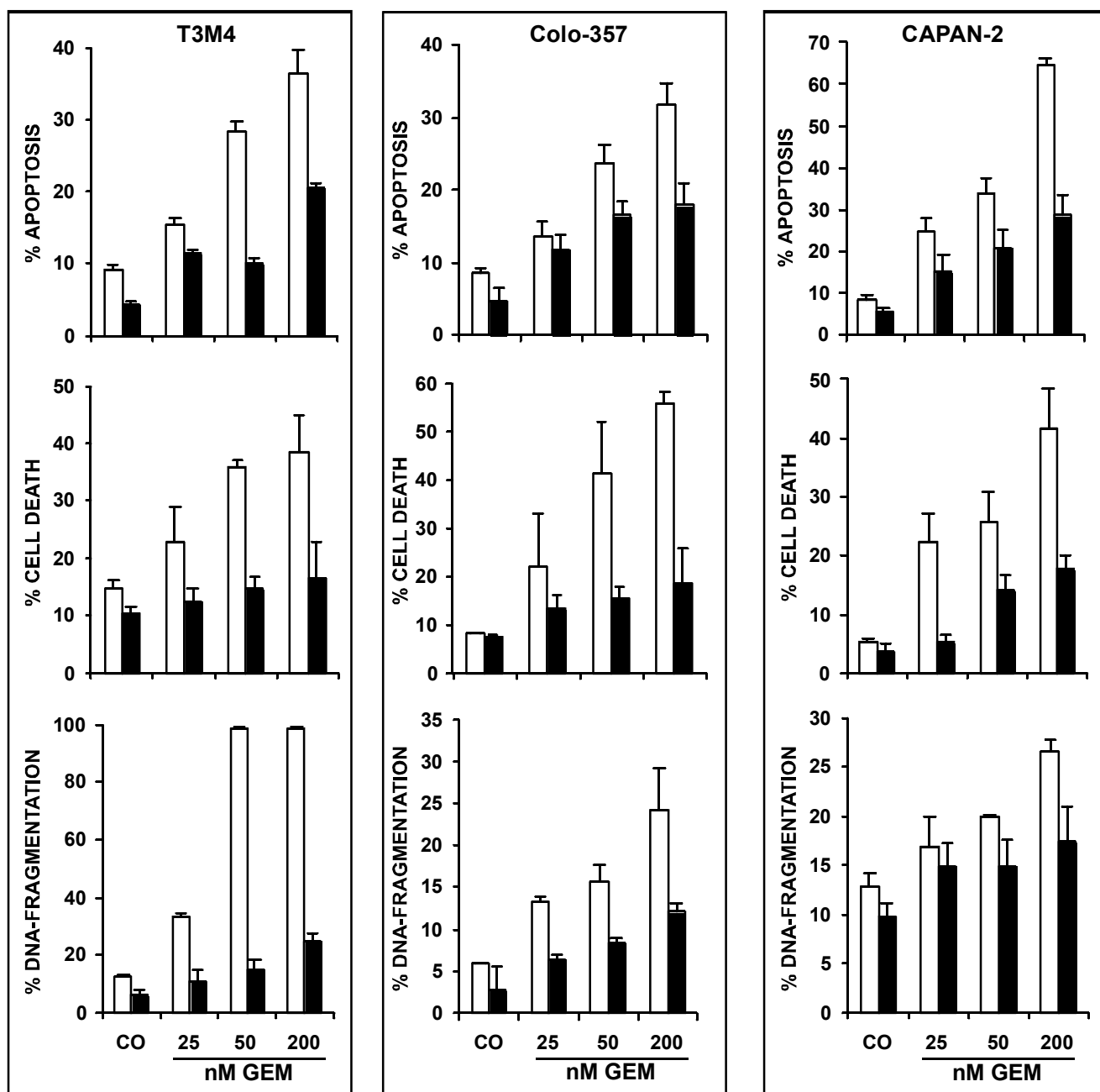

Supplement: Additional File I — DEX inhibits apoptosis in response to gemcitabine in vitro The established pancreatic cancer cells T3M4, Colo-357 and Capan-2 were left either untreated (CO) or were treated with gemcitabine (GEM: 25, 50, 200 μM) in the absence (white bars) or presence (black bars) of DEX (1 μM) which was added 48 h prior to cytotoxic treatment. 72 h following addition of gemcitabine, (A) apoptosis was analyzed by staining of the cells with annexin-FITC (% APOPTOSIS), with nicoletti buffer (% DNA-FRAGMENTATION) and FACS-analysis or cells were left unstained and cell morphology was analyzed by FACS analysis (% CELL DEATH). [file 1471-2407-6-61-S1.pdf]

# Fresh pancreatic carcinoma cells treated with gemcitabine +/- DEX immediately after resection

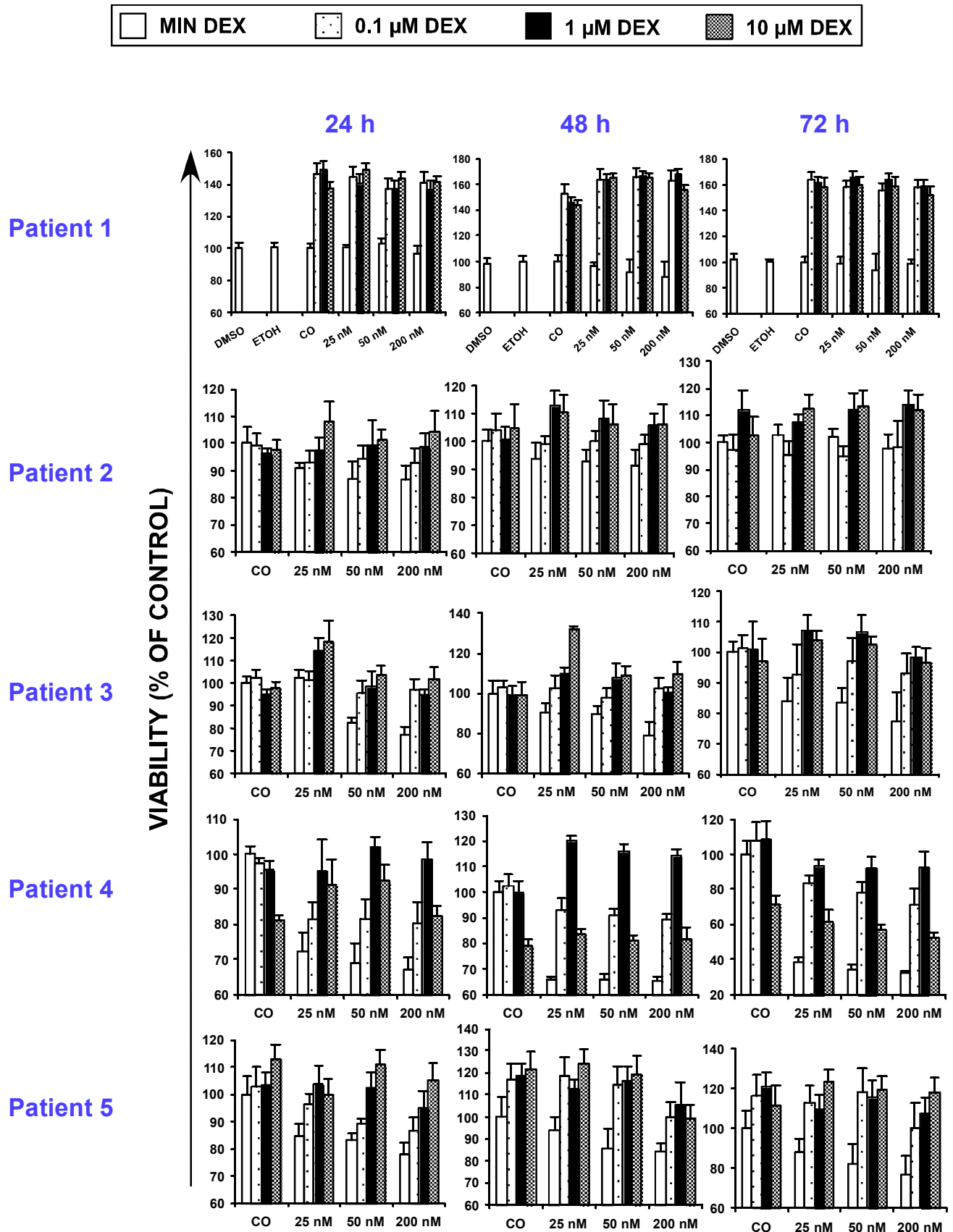

Supplement: Additional File II — DEX induces resistance ex vivo Tumour cells from 20 patients (No. 1-20) with pancreatic cancer were freshly isolated and cultivated in a concentration of 5 × 10^5^/ml in the absence (white bars) or presence of DEX (0.1, 1 or 10 μM as indicated) for 24 h. Gemcitabine or cisplatin were added in concentrations indicated while the controls remained untreated (CO) or were treated with the solvents alone. 24, 48 and 72 h after adding cytotoxic drugs, viability was measured by the MTT-assay. Eight wells per treatment were analyzed and standard deviations are less than 10%. [file 1471-2407-6-61-S2.pdf]

# Fresh pancreatic carcinoma cells treated with gemcitabine +/- DEX immediately after resection

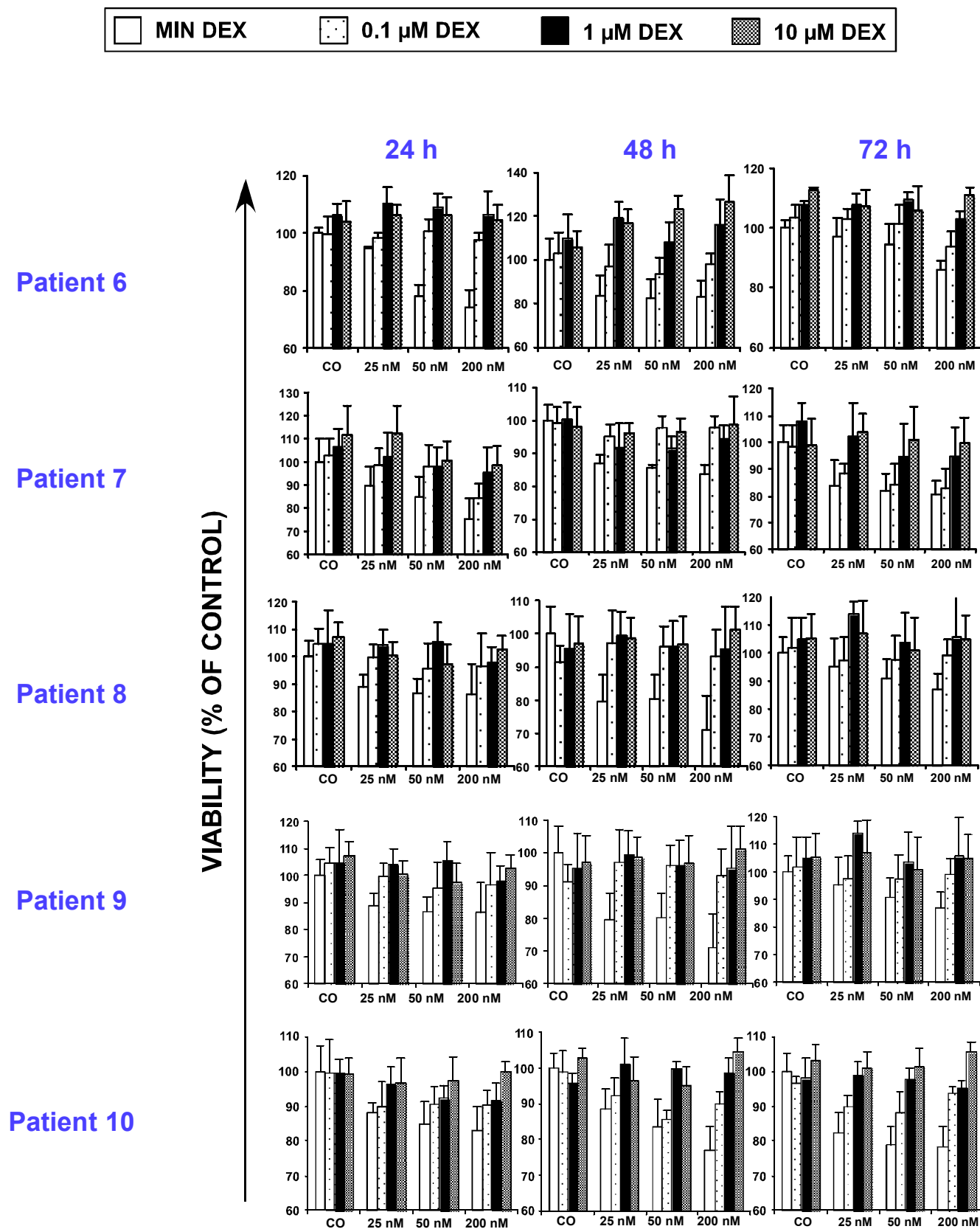

Supplement: Additional File III — DEX induces resistance ex vivo Tumour cells from 20 patients (No. 1-20) with pancreatic cancer were freshly isolated and cultivated in a concentration of 5 × 10^5^/ml in the absence (white bars) or presence of DEX (0.1, 1 or 10 μM as indicated) for 24 h. Gemcitabine or cisplatin were added in concentrations indicated while the controls remained untreated (CO) or were treated with the solvents alone. 24, 48 and 72 h after adding cytotoxic drugs, viability was measured by the MTT-assay. Eight wells per treatment were analyzed and standard deviations are less than 10%. [file 1471-2407-6-61-S3.pdf]

# Fresh pancreatic carcinoma cells treated with cisplatin +/- DEX immediately after resection

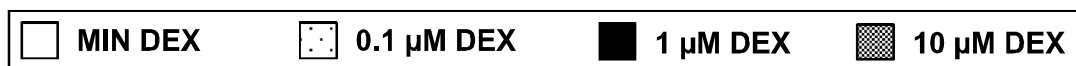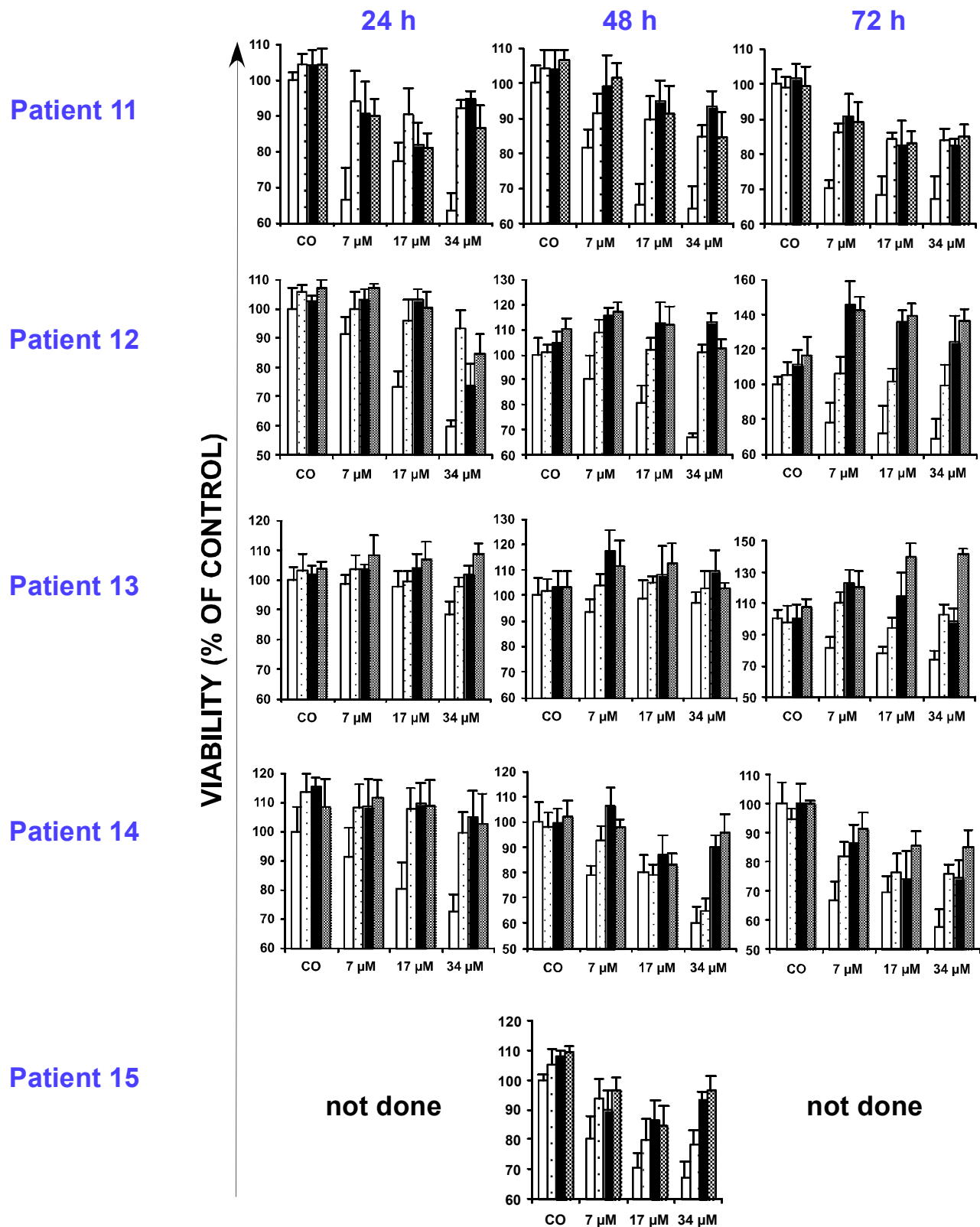

Supplement: Additional File IV — DEX induces resistance ex vivo Tumour cells from 20 patients (No. 1-20) with pancreatic cancer were freshly isolated and cultivated in a concentration of 5 × 10^5^/ml in the absence (white bars) or presence of DEX (0.1, 1 or 10 μM as indicated) for 24 h. Gemcitabine or cisplatin were added in concentrations indicated while the controls remained untreated (CO) or were treated with the solvents alone. 24, 48 and 72 h after adding cytotoxic drugs, viability was measured by the MTT-assay. Eight wells per treatment were analyzed and standard deviations are less than 10%. [file 1471-2407-6-61-S4.pdf]

# Fresh pancreatic carcinoma cells treated with cisplatin +/- DEX immediately after resection

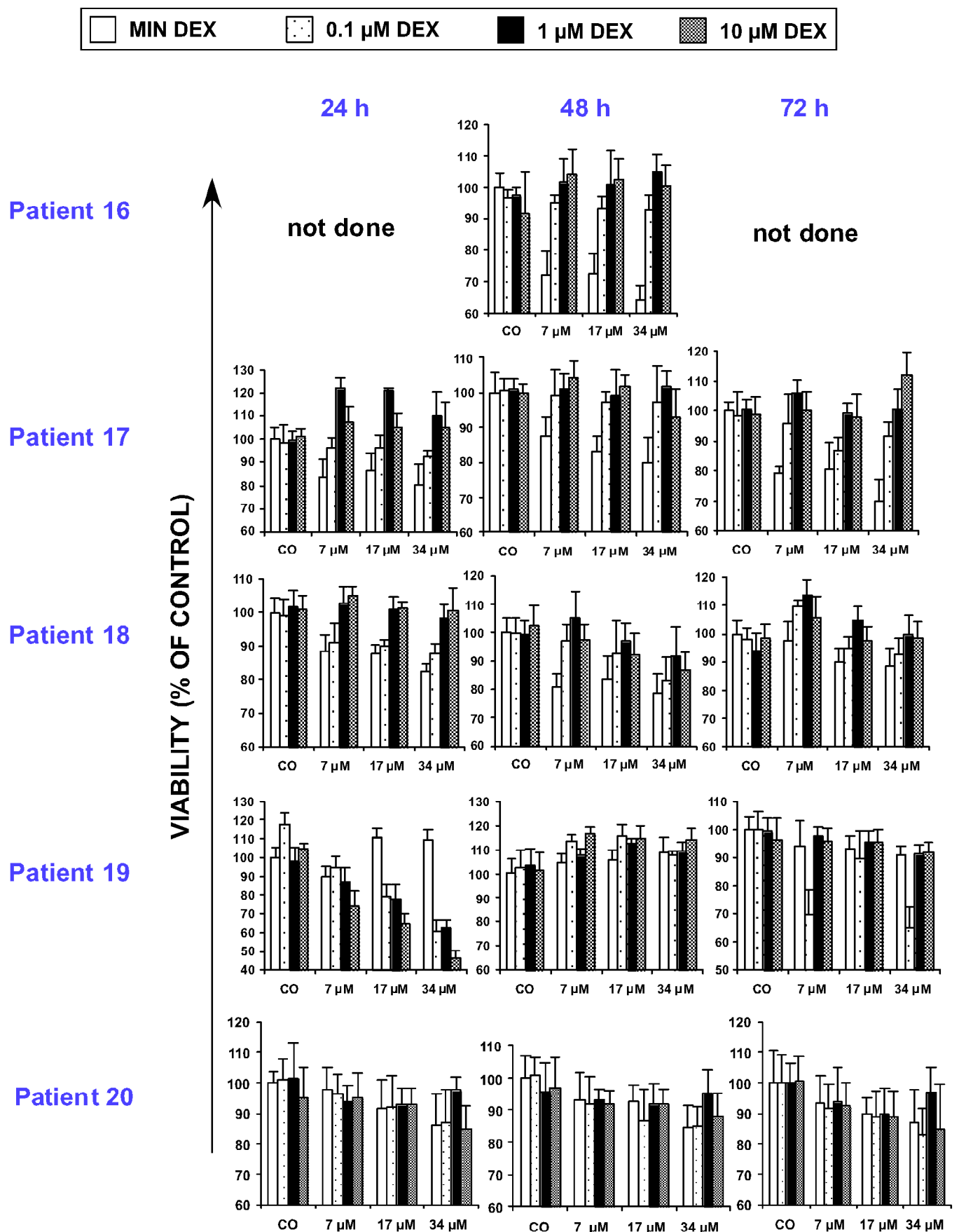

Supplement: Additional File V — DEX induces resistance ex vivo Tumour cells from 20 patients (No. 1-20) with pancreatic cancer were freshly isolated and cultivated in a concentration of 5 × 10^5^/ml in the absence (white bars) or presence of DEX (0.1, 1 or 10 μM as indicated) for 24 h. Gemcitabine or cisplatin were added in concentrations indicated while the controls remained untreated (CO) or were treated with the solvents alone. 24, 48 and 72 h after adding cytotoxic drugs, viability was measured by the MTT-assay. Eight wells per treatment were analyzed and standard deviations are less than 10%. [file 1471-2407-6-61-S5.pdf]
